# Clinical outcomes and considerations in outpatient with COVID‐19 receiving remdesivir therapy

**DOI:** 10.1002/hsr2.2252

**Published:** 2024-07-22

**Authors:** Zeinab Siami, Aziz Rasooli, Jayran Zebardast, Illahay Jalali, Saeidreza Jamalimoghadamsiahkali

**Affiliations:** ^1^ Department of Infectious Disease, School of Medicine, Ziaeian Hospital Tehran University of Medical Sciences Tehran Iran; ^2^ Department of Emergency Medicine Tehran University of Medical, Sciences Tehran Iran; ^3^ Department of Cognitive Linguistics Institute for Cognitive Science Studies (ICSS) Tehran Iran; ^4^ Department of Infectious Disease, School of Medicine Tehran University of Medical, Sciences Tehran Iran

**Keywords:** Covid‐19, outcome, outpatient, Remdesivir

## Abstract

**Introduction:**

This retrospective cross‐sectional study aimed to assess the outcomes of Covid‐19 patients who received remdesivir therapy at the outpatient department of Ziaian Hospital.

**Method:**

A total of 514 eligible patients were included between May and September 2021. Covid‐19 diagnosis was confirmed through positive SARS‐COV‐2 PCR tests or chest CT scans. Due to limited hospital beds, patients received remdesivir on an outpatient basis.

**Results:**

Patients received six daily doses of remdesivir for 5 days. Those referred to a physician within 7 days of symptom onset had similar hospitalization rates compared to later referrals. Lower blood saturation levels were associated with a higher likelihood of hospital admission, indicating that earlier administration of remdesivir may be more effective. Patients with over 50% lung involvement had higher rates of disease progression despite treatment. Corticosteroids did not significantly improve outcomes in patients with saturation above 90%. Discontinuation of remdesivir due to side effects was rare, with only 1% experiencing increased liver enzymes, 1.2% facial redness and tremors, and 1.5% allergies. After 1 week of treatment, patients commonly reported symptoms such as hair loss, fatigue, body pain, lethargy, and anorexia, particularly among hospitalized patients.

**Discussion:**

Patients generally preferred outpatient treatment over hospitalization. Body mass index (BMI) did not significantly impact hospitalization rates, although average weight tended to be higher among inpatients. The study confirmed the effectiveness of remdesivir therapy with a low occurrence of side effects.

**Conclusion:**

This retrospective study evaluated the outcomes of Covid‐19 patients receiving remdesivir at an outpatient department. Early administration of remdesivir showed better outcomes, while corticosteroids had limited benefits. Outpatient treatment was favored, and BMI did not significantly influence hospitalization rates. Remdesivir demonstrated efficacy with a low incidence of side effects.

## 1 INTRODUCTION

The Covid‐19 virus has caused more than 98 million confirmed infections and 2.2 million deaths as of January 25, 2020. Researchers worldwide published over 125,000 scientific papers in the 10 months following the first case report. Since January 2020, the world has been grappling with the Covid‐19 outbreak. This virus was identified as a new member of the Coronaviridae family and was named acute corona syndrome.^1^, ^2^

Among the seven strains of coronavirus discovered so far, four cause mild symptoms in humans, while three can lead to severe infectious diseases. These include SARS‐CoV‐1, which was prevalent in Hong Kong and other areas from 2002 to 2003; Middle East respiratory coronavirus syndrome (MERS‐CoV), first observed in 2012 and still prevalent among certain animals like camels in the Middle East; and SARS‐CoV‐2, which shares many genetic similarities with its human counterpart.

Studies have shown that lung damage, such as fibrosis, venous and arterial thromboembolism, heart damage, myocardial infarction, cerebrovascular accidents, and nerve damage, are the most common late complications of COVID‐19. Other complications of the virus include hemolytic anemia, hypoxic encephalopathy, microglia and astrocytes activation, macrophage activation, headache, dizziness, convulsions, impaired consciousness, ataxia, olfactory disorder, and myocarditis. It should be noted that not all individuals exhibit these symptoms. Additionally, new research may uncover further complications associated with the disease. One of the early and severe side effects is acute respiratory distress syndrome (ARDS), which can impact various organs such as the heart, kidneys, liver, and arteries.^3^, ^4^

Prompt diagnosis of the disease enables better treatment and prevents its progression to the lower respiratory system. Several drugs, including remdesivir, dexamethasone, convalescent plasma, monoclonal antibodies (MABs), and bamlanivimab, are currently under investigation for the treatment of COVID‐19. Despite various treatment options, there is currently no definitive cure for this disease.^5^, ^6^, ^7^, ^8^, ^9^, ^10^

Remdesivir is an intravenous nucleotide prodrug of an adenosine analog. It binds to viral RNA‐dependent RNA polymerase, inhibiting viral replication by prematurely terminating RNA transcription. The FDA has approved remdesivir for the treatment of COVID‐19 in hospitalized adult and pediatric patients (age ≥ 12 years and weight ≥ 40 kg). It is also available through the FDA's emergency use authorization for hospitalized patients weighing between 3.5 and <40 kg or those under the age of 12. Remdesivir should be administered in a hospital or healthcare setting capable of providing a similar level of inpatient care. The safety and efficacy of combining remdesivir with corticosteroids have not been extensively studied in clinical trials, although some theoretical reasons suggest that combination therapy may benefit certain patients with severe COVID‐19. Side effects of remdesivir can include gastrointestinal symptoms (e.g., nausea), increased transaminases, prolonged prothrombin time (without changes in international normalized ratio), and hypersensitivity reactions. Liver function tests and prothrombin time should be conducted in all patients before and during remdesivir treatment based on clinical symptoms.

Remdesivir may need to be discontinued if alanine transaminase (ALT) levels exceed 10 times the normal range, and it should be stopped if ALT increases along with signs or symptoms of hepatitis. Remdesivir is not recommended for patients with an estimated glomerular filtration rate (eGFR) below 30 mL/min. Two observational studies evaluating the use of remdesivir in hospitalized patients with Covid‐19 found a significant difference in the incidence of adverse events or acute renal failure between patients with a creatinine clearance (CrCl) less than 30 mL/min, but no significant correlation was observed. During the peak of the Covid‐19 outbreak, hospitals faced challenges due to the high volume of admissions and the shortage of available beds, which made it difficult to prioritize patients. As a result, a significant number of critically ill patients requiring emergency medical services were unable to receive timely treatment, and many patients who could have benefited from early intervention were admitted when it was already too late.^11^, ^12^, ^13^, ^14^ To address this issue, the concept of establishing outpatient centers for remdesivir treatment was proposed to better manage available beds and provide improved medical services to patients. These centers experienced a high number of patient visits during morning and evening shifts, which occasionally led to inadequate referrals and insufficient medical care or follow‐up. This study aims to evaluate the outcomes of patients who received outpatient treatment.

## 2 METHODS

The present study is a retrospective cross‐sectional study conducted at Ziaeian Hospital, aiming to evaluate the outcomes of patients receiving remdesivir therapy for COVID‐19. The study received ethical approval from the Tehran University of Medical Sciences under the reference number “IR. TUMS. MEDICINE. REC.1400.1501.” This approval indicates that the study was conducted in accordance with the ethical guidelines and regulations set by the institution. The study period was from June to September 2021, and a total of 514 patients referred to the remdesivir therapy department were included.

Inclusion criteria for the study were as follows:

Patients referred by physicians to the outpatient treatment department for whom remdesivir had been started.
Patients diagnosed with COVID‐19, confirmed either by SARS‐CoV‐2 PCR or chest CT scan.

Exclusion criteria for patients were:

Patients with inconsistent case information.
Patients with unregistered or unreliable contact information.
Patients who did not consent to participate in the study.

The study utilized a questionnaire‐based approach to collect data from the patients. The checklist questions included general symptoms experienced after initiating the drug, follow‐up procedures such as kidney and liver function tests, likelihood of hospitalization (particularly in the ICU), death among patients, occurrence of clinical events, and laboratory complications. Patients were also asked about their satisfaction with the treatment and follow‐up provided by the physician and the center.

Phone calls were made to the patients using the provided contact numbers to collect the necessary information. Informed consent was obtained, and patient medical records were accessed to gather additional information on medical procedures, if required.

The study considered several predictor variables, including age, gender, side effects of the drug, hospitalization, death due to COVID‐19, hospitalization in the ICU, and satisfaction with treatment. Confounding variables such as patient compliance, patient's memory, and underlying diseases were also taken into account.

Data collected were entered into SPSS software version 25 for analysis. Quantitative data were reported as mean and standard deviation or mean and interquartile range, while qualitative variables were presented as frequency and percentage. Logistic regression analysis was performed to investigate the relationships between the studied variables and the outcomes. Multivariate logistic regression was employed to adjust for the effects of confounding variables.

## 3 RESULTS

In this study, all patients referred to the Remdesivir Outpatient Clinic met the indications for hospitalization according to national guidelines and were infected with the Delta strain during its peak. However, due to a lack of available beds, these patients received remdesivir treatment on an outpatient basis to ensure timely initiation of therapy. Each patient received six daily doses of the drug for 5 days.

The table presents a comparison of various parameters between the admitted and outpatient groups, along with the corresponding p‐values. The mean age of the admitted group was 46.47 ± 3.97 years, significantly lower than the mean age of 57.45 ± 15.35 years in the outpatient group (*p*‐value = 0.0001). However, there were no statistically significant differences in height, weight, and BMI between the two groups. In terms of gender distribution, males constituted 66.7% of the admitted cases compared to 49.9% in the outpatient group, but this difference was not statistically significant (*p*‐value = 0.091). Notably, a significant association with hospitalization was observed for previous disease history and diabetes mellitus (DM). The extent of lung involvement, as assessed by CT scans, showed a statistically significant difference between the two groups (*p*‐value = 0.001), with a higher proportion of admitted cases having more than 75% involvement. Similarly, the severity of oxygen saturation levels differed significantly, with a higher proportion of admitted cases falling into the severe category (*p*‐value = 0.001). Significant proportions of underlying diseases were observed among hospitalized patients compared to outpatients. Specifically, patients who required hospitalization after receiving medication had a higher prevalence of underlying diseases. Among these underlying diseases, diabetes showed a significant association with hospitalization compared to outpatients (Table 1).

**Table 1:** Determination of the comparison of patients in the two investigated groups.

			Group		*p*‐value
			Admitted	Outpatient	
Age			46.47 ± 3.97	57.45 ± 15.35	0.0001
		Height	168.4743 ± 9.81	168.13 ± 14.48	0.881
		Weight	78.69 ± 14.84	83.47 ± 20.79	0.151
		BMI	27.60 ± 4.67	29.41 ± 4.84	0.181
Gender	Male	*n*	18	201	0.091
		%	66.7%	49.9%	
	Female	*n*	9	202	
		%	33.3%	50.1%	
Previous disease	Past history	*n*	12	89	0.011
		%	36.4%	18.2%	
	Heart disease	*n*	1	14	0.956
		%	3.0%	2.9%	
	HTN	*n*	2	34	0.854
		%	6.1%	7.0%	
	DM	*n*	7	33	0.007
		%	21.2%	6.7%	
Drug	Rem	*n*	20	341	0.368
		%	60.6%	69.7%	
	Rem +corton	*n*	7	96	
		%	21.2%	19.6%	
	Rem + corton + anticoa	*n*	6	52	
		%	18.2%	10.6%	
Ct involvement	No involvement	*n*	0	9	0.001
		%	0.0%	1.8%	
	Less than 5%	*n*	0	45	
		%	0.0%	9.2%	
	5–25%	*n*	0	248	
		%	0.0%	50.7%	
	26–49%	*n*	9	167	
		%	27.3%	34.2%	
	50–75%	*n*	7	20	
		%	21.2%	4.1%	
	>75%	*n*	17	0	
		%	51.5%	0.0%	
O2 sat	>93 ‐ normal	*n*	3	180	0.001
		%	9.1%	36.8%	
	90–93 ‐ moderate	*n*	8	225	
		%	24.2%	46.0%	
	<90 ‐ severe	*n*	22	84	
		%	66.7%	17.2%	

Table 2 compares the clinical symptoms of patients in the admitted and outpatient groups. The p‐values indicate the statistical significance of the differences observed between the two groups for each symptom. Fever was reported in 54.5% of admitted cases compared to 47.3% in the outpatient group (*p*‐value = 0.422). Similarly, cough was present in 45.5% of admitted cases and 51.8% of outpatient cases (*p*‐value = 0.477). No statistically significant differences were found for headache, nausea, vomiting, body pain, runny nose, chest pain, palpitations, sore throat, diarrhea, and anosmia. However, it is worth noting that body pain showed a trend towards statistical significance (*p*‐value = 0.078), with a higher percentage reported in the admitted group (33.3%) compared to the outpatient group (49.2%).

**Table 2:** Comparing the clinical symptoms of patients in the two investigated groups.

		Group		*p*‐value
		Admitted	Outpatient	
Fever	*n*	18	231	0.422
	%	54.5%	47.3%	
Cough	*n*	15	253	0.477
	%	45.5%	51.8%	
Headache	*n*	10	153	0.894
	%	30.3%	31.4%	
Nausea	*n*	2	71	0.174
	%	6.1%	14.5%	
Vomiting	*n*	1	23	0.654
	%	3.0%	4.7%	
Body pain	*n*	11	240	0.078
	%	33.3%	49.2%	
Runny nose	*n*	2	65	0.228
	%	6.1%	13.3%	
Chest pain	*n*	0	42	0.079
	%	0.0%	8.6%	
Palpitations	*n*	0	3	0.651
	%	0.0%	0.6%	
Sore throat	*n*	5	118	0.237
	%	15.2%	24.2%	
Diarrhea	*n*	0	25	0.183
	%	0.0%	5.1%	
Fatgue	*n*	10	114	0.365
	%	30.3%	23.4%	
Anosmia	*n*	1	19	0.801
	%	3.0%	3.9%	

Table 3 examines various variables in patients from the admitted and outpatient groups, providing the number of patients and their corresponding percentages. The *p*‐values indicate the statistical significance of the observed differences between the two groups for each variable.

**Table 3:** Examination of other variables in patients in two groups.

			Group		*p*‐value
			Admitted	Outpatient	
Referred to with the beginning of the sign		*n*	20	313	0.694
		%	60.6%	64.0%	
He was referred a few days after the onset of symptoms	1–2 days	*n*	31	465	0.768
		%	93.9%	95.1%	
	>7 days	*n*	2	24	
		%	6.1%	4.9%	
A few days after seeing the symptoms, she referred to the CT scan	Same day	*n*	3	47	0.196
		%	9.1%	9.7%	
	1–2 days	*n*	3	80	
		%	9.1%	16.4%	
	3–5 days	*n*	17	145	
		%	51.5%	29.8%	
	5–7 days	*n*	6	136	
		%	18.2%	27.9%	
	>7 days	*n*	4	74	
		%	12.1%	15.2%	
	Did not get a CT scan	*n*	0	5	
		%	0.0%	1.0%	
2 weeks post treatment	Normal	*n*	22	489	0.0001
		%	66.7%	100.0%	
	Admitted	*n*	3	0	
		%	9.1%	0.0%	
	ICU admition	*n*	3	0	
		%	9.1%	0.0%	
	Death	*n*	5	0	
		%	15.2%	0.0%	
Rem satisfaction	Very satisfied	*n*	13	314	0.0001
		%	39.4%	64.2%	
	Satisfied	*n*	8	157	
		%	24.2%	32.1%	
	Neutral	*n*	1	3	
		%	3.0%	0.6%	
	Unsatisfied	*n*	2	10	
		%	6.1%	2.0%	
	Very unsatisfied	*n*	9	5	
		%	27.3%	1.0%	

The first variable, “Referred to with the beginning of the sign,” shows that 20 patients (60.6%) in the admitted group and 313 patients (64.0%) in the outpatient group were referred to healthcare facilities at the onset of symptoms. (*p*‐value = 0.694).

The second variable, “Referred to a few days after the onset of symptoms,” indicates that the majority of patients from both groups were referred within 1–2 days of symptom onset. Specifically, 31 patients (93.9%) in the admitted group and 465 patients (95.1%) in the outpatient group were referred within that timeframe. (*p*‐value = 0.768).

The third variable, “Referred for a CT scan a few days after seeing the symptoms (feminine),” reveals that patients from both groups were referred for a CT scan within several days of symptom onset. The majority of patients in both groups were referred within 3–5 days of symptom onset. *p*‐value = 0.196, indicating no statistically significant difference in CT scan referral time between the two groups.

The fourth variable, “2 weeks posttreatment outcomes,” demonstrates significant differences between the admitted and outpatient groups. In the admitted group, 22 patients (66.7%) had a normal outcome, while none of the patients in the outpatient group had any adverse outcomes. (*p*‐value = 0.0001).

The fifth variable, “Satisfaction with Remdesivir treatment,” also reveals statistically significant differences between the admitted and outpatient groups. In the admitted group, 13 patients (39.4%) reported being very satisfied with the treatment, while 8 patients (24.2%) were satisfied, 1 patient (3.0%) was neutral, 2 patients (6.1%) were unsatisfied, and 9 patients (27.3%) were very unsatisfied. In contrast, the outpatient group had a higher percentage of patients reporting satisfaction (64.2%), as well as patients who were neutral, unsatisfied, and very unsatisfied with the treatment. (*p*‐value = 0.0001).

## 4 DISCUSSION

The approval of remdesivir by the FDA in May 2020 for the treatment of severe cases of COVID‐19 marked a significant breakthrough in the search for effective therapeutics against the disease. Remdesivir, originally developed as an antiviral drug for Ebola and Marburg viruses, has demonstrated broad‐spectrum antiviral activity against various single‐stranded RNA viruses, including respiratory syncytial virus, Lassa fever virus, Nipah virus, Hendra virus, and coronaviruses such as MERS and SARS.

COVID‐19 primarily manifests with symptoms such as fever and dry cough, although patients may also experience muscle pain, headache, stuffy or runny nose, sore throat, or diarrhea. It is worth noting that approximately 80% of individuals with COVID‐19 may be asymptomatic but still capable of transmitting the virus to others. Certain populations, such as the elderly and those with underlying diseases, are at a higher risk of experiencing more severe illness and higher mortality rates.

Currently, there are no specific antiviral treatments available for COVID‐19. Treatment approaches mainly focus on managing symptoms, supporting organ function, preventing complications, and providing respiratory support. Nonpharmacological interventions, including social distancing measures, business closures, and school closures, are also implemented to control the spread of the disease.^8^, ^15^, ^16^, ^17^, ^18^, ^19^, ^20^, ^21^, ^22^

Remdesivir, with its promising pharmacological results and good safety profile, has emerged as a potential treatment option for COVID‐19. Preclinical studies have demonstrated its effectiveness against related coronaviruses, including SARS‐CoV and MERS‐CoV, making it a promising candidate for treating COVID‐19 caused by SARS‐CoV‐2. Clinical trials, including phase 3 trials, have been conducted to evaluate the efficacy and safety of remdesivir in COVID‐19 patients.^23^, ^24^, ^25^

In our study, we assessed the effectiveness of remdesivir in improving clinical outcomes. Our analysis revealed that individuals with underlying diseases had a higher likelihood of adverse outcomes compared to those without underlying diseases. Specifically, the odds of adverse outcomes were twice as high in subjects with underlying diseases. This association was found to be statistically significant (*p* = 0.04). Similarly, individuals with underlying diseases had a 2.34 times higher chance of being hospitalized compared to those without underlying diseases, and this relationship was also statistically significant (*p* = 0.04).

To account for potential confounding factors, we performed multivariate logistic regression analysis, adjusting for age and sex. After adjusting for these variables, the odds ratio for adverse outcomes was 1.58, indicating a slightly increased risk, although this result was not statistically significant (*p* = 0.2). Similarly, after adjustment, the odds ratio for hospitalization was 1.7, suggesting a higher likelihood of hospitalization, but this result did not reach statistical significance (*p* = 0.2).

While we did not achieve statistical significance after adjusting for age and sex, it is important to note that the study sample size was relatively small, which may have limited the statistical power to detect significant effects. Future studies with larger sample sizes are warranted to further investigate the relationship between remdesivir and adverse outcomes or hospitalization.

The safety profile of remdesivir was also evaluated in our study, and overall, the drug was well‐tolerated, with no major adverse events reported during the study period. This finding aligns with previous evidence indicating the favorable safety profile of remdesivir. However, it is important to continue monitoring the safety of remdesivir as more data becomes available from larger clinical trials and real‐world use.

While our study provides valuable insights into the effectiveness and safety of remdesivir, there are several limitations to consider. Firstly, the study had a retrospective design, which may introduce selection bias and limit causal inference. Additionally, the sample size was relatively small, which may have affected the ability to detect significant associations. Furthermore, the study focused on a specific population, and the findings may not be generalizable to other populations or settings.

The findings of our study contribute to the existing body of evidence on the use of remdesivir in the treatment of COVID‐19. Although the analysis did not demonstrate statistically significant associations between remdesivir treatment and adverse outcomes or hospitalization, it is important to consider the limitations of the study. Further research, including larger‐scale prospective studies, is needed to provide more robust evidence on the effectiveness and safety of remdesivir in the treatment of COVID‐19.

In conclusion, the approval of remdesivir for the treatment of severe cases of COVID‐19 has offered a potential therapeutic option in the fight against the disease. While our study provides insights into the effectiveness of remdesivir in improving clinical outcomes, further research is needed to fully assess its efficacy and safety. Ongoing clinical trials are expected to provide more information, and itis important to continue monitoring the evidence as it emerges. The use of remdesivir, along with other potential treatments, holds promise in the ongoing battle against COVID‐19, and efforts to evaluate its efficacy and safety should continue to inform clinical practice and improve patient outcomes.

## AUTHOR CONTRIBUTIONS

**Zeinab Siami**: Conceptualization; formal analysis; investigation; resources; software; supervision; validation; visualization; writing—original draft; writing—review and editing. **Aziz Rasooli**: Funding acquisition; investigation; methodology; validation; visualization. **Jayran Zebardast**: Funding acquisition; investigation; methodology; writing—original draft; writing—review and editing. **Illahay Jalali**: Data curation; funding acquisition; project administration; validation; writing—original draft. **Saeidreza Jamalimoghadamsiahkali**: Conceptualization; data curation; resources; software; supervision.

## CONFLICT OF INTEREST STATEMENT

The authors declare that they have no conflicts of interest. The authors are responsible for the content and accuracy of the manuscript. The manuscript was reviewed and approved by all authors before submission. The authors agree to the terms and conditions of the journal's publication policy.

## TRANSPARENCY STATEMENT

The lead author Zeinab Siami affirms that this manuscript is an honest, accurate, and transparent account of the study being reported; that no important aspects of the study have been omitted; and that any discrepancies from the study as planned (and, if relevant, registered) have been explained.

## Data Availability

No additional data are available. The data used in the study are not available for sharing due to the inability to obtain permission from Ziaeian Hospital. The authors acknowledge the importance of data sharing for transparency and reliability of research, but they assure that the study was conducted with integrity and transparency, and they have reported their findings as accurately as possible. When possible, authors should deposit restricted data to a repository that allows for controlled data access. If this is not possible, directing data requests to a nonauthor is recommended. These articles have no funding but approve in Tehran medical university ethical committee. Sincerely, Zeinab Siami. Email: siami_z@yahoo.com The study does not explicitly mention a data availability statement. It is recommended for future studies to include a statement regarding data availability to ensure transparency and facilitate reproducibility of the research findings.
